# 2011–2012 Reviewers

**DOI:** 10.1002/brb3.121

**Published:** 2013-01-09

*Brain and Behavior* would like to thank all of the reviewers whose support has led to the successful and continuing growth of the journal. We rely on the expertise and judgment of our reviewers and appreciate the time and effort put into these reviews. We also would like to thank the numerous reviewers at our supporter journals whose reviews were passed along via the manuscript transfer program for their hard work. The manuscript transfer program has a goal of reducing the burden on working researchers to complete reviews by sending the previous reviewer comments of papers transferred to *Brain and Behavior*, creating a seamless peer-review process. Below is a list of the reviewers submitting one or more reviews directly to *Brain and Behavior* between March 2011 and December 2012.

Addis, Donna Rose

Alford, Simon

Amrein, Hubert

Anagli, John

Apkarian, A.Vania

Arampatzis, Adamantios

Aronowski, Jarek

Aslund, Cecilia

Atchley, Ruth Ann

Ayalon, Liat

Barlinn, Kristian

Bashir, Khurram

Bauer, Michael

Bavarsad, Reza

Belin, David

Benzel, Edward

Bergeron, Marcelle

Berryhill, Marian

Bolshavkov, Vadim

Brown, Jesse

Bryan, Robert

Burman, Doug

Caplan, Lou

Carmin, Cheryl

Carre, Justin

Caudle, Robert

Center, Kristy

Chabbert, Christian

Chatelle, Camille

Cherbuin, Nicolas

Chiariotti, Lorenzo

Choi, Sang-Ho

Chorlian, David

Ciobanu, Mariana

Clark, David

Cooper, Joseph

Crews, Fulton

Cruse, Damian

Darriba, Alvaro

Dassonville, Paul

Davies, Kay

De Ridder, Dirk

DeGrush, Elizabeth

Demertzi, Athena

Devous, Michael

Diamond, David

Dick, Finlay

Domino, Edward

Dorval, Alan Chuck

Ehlers, Cyndy

Elliott, Glen

Espinoza, Randall

Ettinger, Ulrich

Falkai, Peter

Fazan Jr., Rubens

Fears, Scott

Feigin, Andrew

Feldmann, Ed

Garcia, Michael

Gard, Paul

Gathercole, Susan

Genova, Helen

Giacobini, Paolo

Giannopoulos, Sotirios

Glanzman, David

Goldberg, Joseph

Greene, Deanna

Griffith, Kathleen

Grimshaw, Gina

Groh, Janos

Grunwald-Kadow, Ilona

Guerard, Katherine

Hald, Lea

Hall, Jeremy

Hamilton, Bruce

Hamilton, John

Han, Xiaosi

Harris, R Adron

Havton, Leif

Hegerl, Ulrich

Hess, David

Ho, Shu-Leong

Hol, Elly M.

Horvath, Tamas L.

Hoyer, Carolin

Huber, Robert

Huber Brösamle, Andrea

Huyck, Julia

Iacoboni, Marco

Iizuka, Takahiro

Ikeda, Masayoshi

Ikenaka, Kazuhiro

Jaarsma, Dick

Jaitner, Thomas

Janssen, Ben

Jolij, Jacob

Karlsgodt, Katherine

Kelly, Kevin

Kerr, Wesley

Kesner, Raymond

Kobak, Ken

Kosel, Markus

Koster, Ernst

Kotrotsiou, Evagelia

Kouvelas, Elias

Kovelman, Ioulia

Kraemer, Helena

Krebs, Ruth

Kuba, Miroslav

LaDu, Mary Jo

Lamont, Scott

Lau, Ellen

Lee, Jonathan

Lenartowicz, Agatha

Li, Jianrong

Lysakowski, Anna

Magnuson, David

Marshak, Sonya

Masten, Carrie

McAndrews, Mary Pat

McCullough, Louise

McDermott, Rose

McGill, Trevor

McNay, Ewan

Mendez, Mario

Moreira, Paula

Mortazavi, Martin

Mouzas, Odiseas

Muller, Yunhua

Naeem, Muhammad

Nagy, Mohamed

Naït-Ali, Amine

Naus, CC

Navarro, Jose

Navarro, Xavier

Newell, David

Newman, Sharlene

Newton, Thomas

Okun, Michael

Olsen, Richard

Olson, James

Olson, Eric

Onocko-Campos, Rosana

Opere, Catherine

Ostrovsky, Yuri

Otten, Leun

Paskavitz, James

Paynter, Christopher

Peng, Junmin

Pereira, Frederico

Perkins, Adam

Perrin, Florence Evelyn

Petteri Piepponen, Timo

Pezet, Sophie

Pilowsky, Paul

Pitts, Michael

Polak, Joseph

Prado, Jerome

Price, Matthew

Psarropoulou, Caterina

Rada, Pedro

Rae, Charlotte

Raedt, Robrecht

Ramboz, Sylvie

Ran, Mao-Sheng

Reder, Lynne

Rende, Richard

Reverberi, Carlo

Roche-Labarbe, Nadège

Rochkind, Shimon

Roman, Gustavo

Rosas, Diana

Rubin, Robert

Ruotolo, Francesco

Saab, Sammy

Sabb, Fred

Salvaris, Matthew

Sass, Katharina

Savine, Adam

Scheiermann, Christoph

Schmid, Petra

Shahed, Joohi

Sharma, Vijay

Sheikh, Kazim

Shinkareva, Svetlana

Shontaz, Edran

Shprecher, David

Simanova, Irina

Singhal, Aneesh

Sjöberg, Rickard

Solet, Jo

Sonntag, Kai

Southgage, Victoria

Spinner, Robert

Spreng, Nathan

Steiger, Axel

Stevens, Beth

Stopková, Pavla

Sumaviella, Teresa

Sunderland, Matthew

Takahata, Masakazu

Thiel, Christiane

Tierney, Ann

Tsirka, Stella

Tsivgoulis, George

Valdois, Sylviane

van Erp, Theo

van Waarde, Jeroen A.

Vaphiades, Michael

Vidyasagar, Dharmapuri

Viviani, Barbara

Walker, Harrison

Wasaka, Toshiaki

Wascher, Edmund

Wenjie, Ren

Wilmouth, Carrie

Wise, Thomas

Wismeijer, Dagmar

Yassa, Michael

Yelnik, Alain

Yi, Chun-Xia

Yonelinas, Andrew

Zimmer, Andreas

